# Application of the Doylestown algorithm for the early detection of hepatocellular carcinoma

**DOI:** 10.1371/journal.pone.0203149

**Published:** 2018-08-31

**Authors:** Anand S. Mehta, Daryl T.-Y. Lau, Mengjun Wang, Aysha Islam, Bilal Nasir, Asad Javaid, Mugilan Poongkunran, Timothy M. Block

**Affiliations:** 1 Department of Cell and Molecular Pharmacology and Experimental Therapeutics, Medical University of South Carolina, Charleston, South Carolina, United States of America; 2 Liver Research Center, Beth Israel Deaconess Medical Center, Harvard Medical School, Boston, Massachusetts, United States of America; 3 The Baruch Blumberg Institute, Doylestown, Pennsylvania, United States of America; 4 The Hepatitis B Foundation, Doylestown, Pennsylvania, United States of America; Kaohsiung Medical University Chung Ho Memorial Hospital, TAIWAN

## Abstract

**Background:**

We previously developed a logistic regression algorithm that uses AFP, age, gender, ALK and ALT levels to improve the detection of hepatocellular carcinoma (HCC). In 3,158 patients from 5 independent sites, this algorithm, referred to as the “Doylestown” algorithm, increased the AUROC of AFP 4% to 12% and had equal benefit regardless of tumor size or the etiology of liver disease.

**Aims:**

Analysis of the Doylestown algorithm using samples from individuals taken before their diagnosis of HCC.

**Methods:**

Here, the algorithm was tested using samples at multiple time points from (a) patients with established chronic liver disease, without HCC (120 patients) and (b) 116 patients with HCC diagnosis (85 patients with early stage HCC and 31 patients with recurrent HCC), taken at the time of, and up to 12 months prior to cancer diagnosis.

**Results:**

Among patients who developed HCC, comparing the Doylestown algorithm at a fixed cut-off to AFP at 20 ng/mL, the Doylestown algorithm increased the True Positive Rate (TPR) in identification of HCC from 36 to 50%, at a time point of 12 months prior to the conventional HCC detection. Similar results were obtained in those patients with recurrent HCC, where the Doylestown algorithm increased TPR in detection of HCC from 18% to 59%, at 12 months prior to detection of recurrence.

**Conclusions:**

This algorithm significantly improves the prediction of HCC by AFP alone and may have value in the early detection of HCC.

## Introduction

Hepatocellular carcinoma (HCC), is often the associated with chronic hepatitis B virus (HBV), chronic hepatitis C virus (HCV) infection, excessive alcohol consumption and nonalcoholic steatohepatitis (NASH) [1]. HCC is the second leading cause of cancer-related death worldwide and the leading cause of death in patients with cirrhosis[1]. Prognosis for patients with HCC is related to tumor stage at time of diagnosis, and there are higher rates of curative treatment and better overall survival among those with early stage tumors[2]. Therefore, HCC surveillance has been recommended for “at-risks patients using ultrasonography, in both antiviral treatment naïve and experienced patients[3]. Surveillance can be performed with or without serum levels of the oncofetal glycoprotein, alpha-fetoprotein (AFP)[4,5]. There has been extensive debate about the utility of AFP, given its suboptimal sensitivity and specificity[6–8]. On the other hand, AFP is an economical and readily available test in both developed and developing countries.

We have recently developed a logistic regression algorithm that utilizes AFP, age, gender, alkaline phosphatase (ALK) and alanine aminotransferase (ALT) levels to improve the detection of HCC, particularly for those with a background of liver cirrhosis[9]. We define this as the Doylestown algorithm. In an analysis of 3,158 patients from 5 independent sites, this algorithm improved detection of HCC as compared to AFP [9]. However, in that previous work, only patients with either cirrhosis or HCC at the time of cancer detection were analyzed. In the present study, we further evaluate the predictability and accuracy of the algorithm by applying it to data from multiple time points from patients with chronic hepatitis without HCC, and from those with cirrhosis before and up to the time of their diagnosis of HCC.

## Materials and methods

### Study populations

For the non HCC subjects, electronic health data were used to randomly select ambulatory clinic patients with chronic liver disease at Beth Israel Deaconess Medical Center (BIDMC), Harvard Medical School. Patients were excluded if they have less than 12 months of clinical observation or if there was suspicious liver mass or HCC identified prior to study enrollment. In addition, only those patients who had the required components of the Doylestown algorithm (age, gender, ALT, ALK and AFP) were utilized in this study. All patients with chronic hepatitis B virus, regardless of cancer status, were on anti-viral therapy (entecavir (Baraclude), tenofovir (Viread), or telbivudine (Tyzeka)). Patients with HCC were identified using ICD9 codes and patient lists of the weekly multidisciplinary HCC conference at BIDMC. All the cases were adjudicated to confirm they met HCC criteria based on the AASLD practice guideline[10]. Patients with tumor size greater then 5 cm at the time of initial HCC diagnosis were excluded from the analysis. As stated, for both the controls and patients with HCC, only patients with available AFP and liver panel (age, gender, ALK and ALT) values at least every 6 months were included.

Briefly, the diagnosis of HCC was made based on accepted standard criteria for one of the following modalities: histopathology, magnetic resonance imaging [MRI], computed tomography (CT) and in a single case, magnetic resonance cholangiopancreatography (MRCP)[10]. Diagnosis of cirrhosis was based on liver histology, Fibroscan score or clinical, and imaging features. For cirrhotic patients to serve as controls, they must have no evidence of HCC by baseline US or MRI of the liver if the AFP was elevated within 3 months prior to enrollment, and for another 6 months after enrollment. The controls in this cohort have been followed for a mean period of 61.6 months (range of 12.7–170.6). Tumor staging was determined using the United Network of Organ Sharing-modified TNM staging system for HCC and 85 (out of 115 possible patients) with early stage HCC were included in this study. Early HCC was defined as T1 (single lesion < 2 cm in diameter) or T2 (single lesion between 2 and 5 cm in diameter) lesions, which met criteria for liver transplantation in the United States.

Patients with recurrent HCC who had at least 12 months of clinical data prior to their cancer diagnosis were also included in this study. Patients were identified systematically and consecutively from the BIDMC electronic HCC registry. In this case, all HCC tumor sizes and stages were included. The detection of HCC was as above. The research study was performed under a BIDMC IRB approved protocol.

Serum AFP and lab values for ALT, ALK were determined using commercially available immunoassays at clinical BIDMC laboratory and taken prior to analysis using the Doylestown algorithm. The data for all patients are found as supplementary data (S1 File).

### Statistical methods

The Doylestown algorithm is comprised of age, gender, log transformation (on base 10) AFP(logAFP), ALK and ALT values. The logistic regression equation was as follows to generate the output value:

1/(1+EXP(-(-10.307 +(0.097*Age)+(1.645*Gender)+(2.314*logAFP)+(0.011*ALK)+(-0.008*ALT)))).

The output value is a continuous variable ranging from 0 to 1. In our prior work an output value of >0.5 was used to identify patients with HCC and had a specificity of >90%[9]. The selection of patients, data collection and application of the algorithm, as found in a base Microsoft Excel file, were uniformly performed at BIDMC.

Descriptive statistics were used. For statistical analysis among groups, in the cases of longitudinal data, linear mixed effects model was applied to compare values among the multiple time points. Kruskal-Wallis One-way ANOVA test was used for independent multiple groups. All analysis applied with R software(www.r-project.org) and GraphPad Prism 5.0 (GraphPad Software, La Jolla, CA, CA).

For comparisons between the sensitivity of AFP and the Doylestown algorithm, We utilized the two sample proportion test to check statistical difference between the true positive rate of AFP and the Doylestown algorithm. The power calculation for each time point followed the guide of Cohen et al 1988 [11].

#### Ethical issues

This study was approved by the Institutional Review Boards of the Beth Israel Deaconess Medical Center, Harvard Medical School.

## Results

### Performance of the Doylestown algorithm in those with chronic liver disease

**Fig 1:**
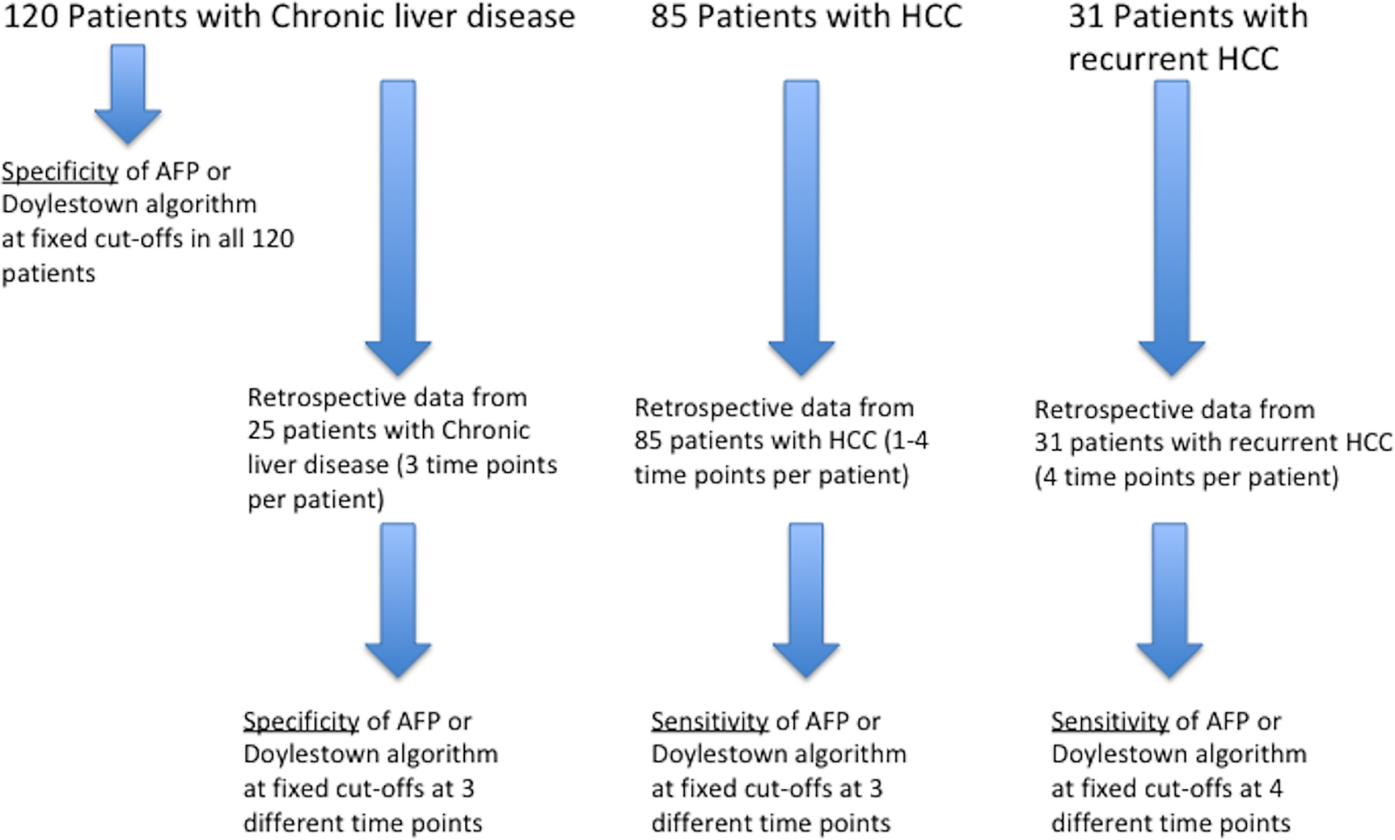
Study design. A total of 236 patients were utilized for this study. 120 without HCC and 116 with HCC. Retrospective longitudinal data was available from 25 patients without HCC and from 116 patients with HCC. In cases without HCC, the specificity of the Doylestown algorithm was compared to AFP at fixed cut-offs. In cases of HCC, the sensitivity of the Doylestown algorithm was compared to AFP at fixed cut-offs.

In our previous work, we analyzed the performance of the Doylestown algorithm in individuals with a diagnosis of cirrhosis or HCC at the time of serum sample collection[9]. However, it was unclear how well this equation would perform with samples from those with chronic liver disease, but without liver cirrhosis or HCC. In addition, it was of interest to determine how early, prior to a diagnosis of HCC, this equation would predict the cancer. To that end, we applied the Doylestown algorithm to data from 120 “control” patients with chronic liver disease (see Table 1) but without cirrhosis or HCC (see Fig 1 for study design). As shown in Table 1, the mean age of these patients was 44.4 with a range of 20 and 82 years of age. The majority of patients were male (56%) and had mean values of ALK, ALT and AFP within the normal range (see Table 1). S1 Fig shows the distribution of AFP (Fig A in S1 Fig) and the Doylestown algorithm (Fig B in S1 Fig) in the control patients and the HCC patients at time zero respectively. Application of the Doylestown algorithm on the sample set of controls yielded a mean output value of 0.07. We have previously determined that Doylestown algorithmic values greater than 0.50 is associated with a diagnosis of HCC.

**Table 1:** Clinical characteristics of control patients without HCC^1^.

Number of patients	120
Gender (% male) ^2^	67/120(56.0%)
Age (Range, years) ^3^	44.4 (20–82)
ALK (Range, U/L)^4^	66.4(26–124)
ALT (Range, U/L) ^5^	41.6(11–350)
AFP (Range, ng/mL) ^6^	3.5 (1.1–34.6)
Duration of Follow Up (Months) ^7^	61.6(12.7–170.6)
Etiology of liver disease: N (%)^8^ HBV HCV NAFLD Alcoholic Liver Disease Autoimmune Hepatitis Mixed liver diseases	92/120 (76%) 15/120 (13%) 4/120 (3%) 1/120 (1%) 1/120 (1%) 7/120 (6%)

1) Patients with chronic liver disease, without evidence of liver cirrhosis.

2) Percentage of male patients.

3) Mean age of patients (in years) with the range indicated.

4) Mean level of alkaline phosphatase (Units per liter (U/L) with the range indicated.

5) Mean level of alanine aminotransferase (U/L) with the range indicated.

6) Mean level of alpha feto protein (ng/mL) with the range indicated.

7) Mean number of months of follow up

8) Etiology of disease, hepatitis B virus, (HBV) hepatitis C virus (HCV), non alcoholic steato hepatitis (NASH) or Non alcoholic fatty liver disease (NAFLD), alcohol associated liver disease, autoimmune hepatitis or patients with mixed liver disease such as HCV/HBV co-infection or HBV in the background of NAFLD.

Therefore, although most of the 120 patients in the control group were properly classified as not having a diagnosis of HCC, two patients in this control group were incorrectly classified (i.e. Doylestown algorithm output as having HCC with cut-off value >0.5). Both of these patients were older, being 65 and 82 years of age, and had AFP <10 ng/mL. One patient (male aged 65) had “normal” levels of AFP (8.9 ng/ml), ALK (67 IU/L) and ALT (38 IU/L). The oldest patient (female aged 82) also had normal levels of AFP (4.5 ng/ml), ALK (94 IU/L) and ALT (12 IU/L).

Longitudinal data was available from 25 chronic HBV infected patients at time 0 [last follow up], 6 months and 12 months prior (a total of 75 samples). A scatter plot of the three main components of the Doylestown algorithm (AFP, ALK and ALT) along with the output values for the Doylestown algorithm are shown in S2 Fig. The mean values of AFP, ALK and ALT are listed in Table 2. As Table 2 shows, for these controls, ALK, ALT and AFP values remained similar over a period of 12 months. As these three factors are key components of the Doylestown algorithm, not surprisingly, the values of the Doylestown algorithm remain low and constant over the time points (Fig D in S2 Fig). One of these 25 patients without HCC had AFP elevation (Fig C in S2 Fig). This patient had an AFP of 32.6 ng/mL at time point 0, 30 ng/mL at 6-month and 28 ng/mL at 12-month prior. The corresponding Doylestown algorithm output values were 0.34, 0.30 and 0.30, all below the 0.5 cut-off for HCC[9]. Importantly, as Table 2 shows, the fluctuations in ALK and ALT values had little impact on the Doylestown algorithm. None of these 25 patients had Doylestown algorithm values ≥ 0.5 (Fig D in S2 Fig).

**Table 2:** Individual component and the Doylestown algorithm at different time-points in patients with chronic liver disease but no cirrhosis and no HCC (n = 25)^1^.

	Individuals followed for 12 months with Liver Disease but no HCC or liver cirrhosis. Time, in months from first collection to last collection			
Analyte	Time 0^2^	Time N6^3^	Time N12^4^	*P value*^*5*^
ALK (range, U/L) ^**6**^	67.1 (34–106)	63.6 (33–117)	64.9 (35–114)	0.721
ALT (range, U/L) ^**7**^	29.1 (16–62)	28.3 (13–65)	28.8 (3–115)	0.585
AFP (range, ng/mL) ^**8**^	3.7 (1.2–32.6)	3.8 (1.5–29.5)	3.7 (1.3–28.0)	0.995
Doylestown algorithm (range) ^**9**^	0.05 (0.0–0.34)	0.05 (0.0–0.30)	0.05 (0.0–0.30)	0.917

1) A total of 75 samples consisting of 3 time points each from 25 individual patients were examined.

2) Time 0 is the time of last data collection.

3) Time N6 is 6 months prior.

4) Time N12 is 12 months prior.

5) Linear mixed effect model.

6) Mean level of alkaline phosphatase (ALK) with the range indicated (in U/L).

7) Mean level of alanine aminotransferase (ALT) with the range indicated (in U/L).

8) Mean level of alpha-fetoprotein (AFP) with the range indicated (in ng/mL).

9) Mean level of the Doylestown algorithm with the range indicated.

### Performance of the Doylestown algorithm in those with HCC

We previously examined the performance of the Doylestown algorithm only in those at the time of HCC diagnosis. Here we have evaluated the performance of the Doylestown algorithm in early HCC detection longitudinally (see Fig 1 for study design). A total of 85 patients with clinical data available from 1 month to 12 months before the diagnosis of HCC were examined (Table 3). The mean size of the tumor at time of diagnosis was 2.8 cm (0.9–4.9 cm). A scatter plot of the three main components of the Doylestown algorithm (AFP, ALK and ALT) along with the output values for the Doylestown algorithm are shown in S3 Fig. The mean values of AFP, ALK and ALT are shown in Table 4. An analysis of the clinical data at the various time points shows that the levels of ALK and ALT do not appreciably change in these individuals as a function of time, similar to that observed with controls. Patients with cirrhosis have much higher levels of ALK and ALT compared to controls (Tables 1 & 3). As expected, AFP levels increase close to the time of cancer diagnosis though the change was not statistically significant (*p* = 0.067). Importantly, the mean Doylestown algorithm output values were >0.5 at time points 9 months prior to HCC diagnosis(*p* = 0.067). Therefore, the algorithm was able to identify patients who ultimately developed HCC as early as 9 months prior to the HCC Diagnosis.

**Table 3:** Clinical characteristics of HCC patients^1^.

Number of patients (N) ^2^	85
Diagnosis of HCC (MRI, CT, Biopsy, MRCP) ^**3**^	42:11:31:1
Mean size of tumor (range, cm) ^**4**^	2.8 (0.9–4.9)
Male (%)^**5**^	72 (84.7%)
Age (Range, years) ^**6**^	62.1 (41.5–87.7)
ALK (Range, U/L) ^**7**^	123.6 (26–582)
ALT (Range, U/L) ^**8**^	74.9 (12–378)
AFP (Range, ng/mL) ^**9**^	8101 (1.1–505,160)
Etiology of disease: N (%)^**10**^	
HBV	10(11.8%)
HCV	38(44.7%)
HBV/HCV Co-infection	1(1.20%)
Alcohol	8(9.0%)
NASH- NAFLD	8(9.0%)
Hemochromatosis	3(3.5%)
Cryptogenic	6(7.0%)
Mixed	11(13.0%)
Cirrhosis N (%)^**11**^	84 (99%)

1)Characteristics of the HCC samples used in this study.

2) The total number of patients examined in this experiment. 1–4 samples were available per patient.

3)Diagnostic methods used for the detection of HCC.

4) Mean size of tumor with the range indicated. All patients are classified as stage 1 or 2 using the UNOS staging system.

5) Percentage of male patients within each group.

6) Mean age of patients with the range indicated.

7) Mean level of alkaline phosphatase (Units per liter (U/L) with the range indicated.

8) Mean level of alanine aminotransferase (U/L) with the range indicated.

9) Mean level of alpha feto protein (ng/mL) with the range indicated.

10) Etiology of disease, hepatitis B virus, (HBV) hepatitis C virus (HCV), HBV/HCV co-infected, alcohol, non alcoholic steato hepatitis (NASH) or Non alcoholic fatty liver disease (NAFLD), Hemochromatosis, Cryptogenic or mixed (excluding HCV/HBV).

11) Patients with biopsy confirmed cirrhosis.

**Table 4:** Individual component and the Doylestown algorithm at different time-points in patients with HCC.

	Time before HCC detected ^1^			
Analyte	1–3 months (N = 104)	6–9 month (N = 52)	12 months (N = 35)	P value^2^
ALK (range,U/L) ^**3**^	130.3 (26–747)	116.6 (50–243)	126.3 (35–360)	0.988
ALT (range,U/L) ^**4**^	81.7 (9–378)	60.6 (13–259)	50.9 (8–199)	0.086
AFP (range,ng/mL) ^**5**^	11,643 (1.1–505,160)	37.0 (1.0–627)	30.0 (1.7–196)	0.016
Doylestown algorithm (range) ^**6**^	0.63 (0.001–1.0)	0.60 (0.13–1.0)	0.58 (0.14–1.0)	0.967

1) Time before detection of HCC, either 1–3 months prior, 6–9 months prior or 12 months prior.

2) Kruskal-Wallis One-way ANOVA test.

3) Mean level of alkaline phosphatase with the range indicated (in U/L).

4) Mean level of alanine aminotransferase with the range indicated (in U/L).

5) Mean level of alpha-fetoprotein with the range indicated (in ng/mL).

6) Mean level of the Doylestown algorithm with the range indicated.

S4 Fig presents the receiver operator characteristic curves (ROC) for AFP or the Doylestown algorithm at either 1–3 months prior to HCC diagnosis (Fig A in S4 Fig, 6–9 months prior to HCC diagnosis (S4B Fig) or 12 months prior to HCC diagnosis (Fig C in S4 Fig). As this figure shows, increases in AUC were observed at all time points as were increased sensitivities at all relevant specificity values. In Table 5, the performance of the Doylestown algorithm was compared to AFP using an output value of ≥0.5 for the Doylestown algorithm and 20 ng/mL for AFP as cutoffs. In our prior analysis, an output of ≥0.5 was associated with HCC with a specificity of ≥90%[9]. The 20 ng/mL cut-off value of AFP was used as historically this has been the accepted level of HCC detection and in one of the largest studies conducted by the National Cancer Institute was associated with 90% specificity[12–15]. Using these values, at 12 months prior to HCC diagnosis, AFP classified 36% of individuals as having HCC and the Doylestown algorithm increased this predictive value to 50%. However, this difference did not meet statistical significance (p = 0.2250) At 6–9 months prior to HCC diagnosis, AFP classified 30% of individuals as having HCC and the Doylestown algorithm increased this rate to 50% (p = 0.036). At 1–3 months prior to HCC detection, AFP identified 58% of those with HCC and the Doylestown algorithm identified 71% (p = 0.0550). Thus, consistent with our previous data, improved performance as compared to AFP was observed with the application of the Doylestown algorithm. The impact of altering the cut-off for AFP or the Doylestown algorithm output value on HCC detection is shown in Table 5. As this table shows, decreasing the cut-off to 10 ng/mL for AFP or to 0.25 for the Doylestown algorithm increased the detection of HCC for both markers. However, as Table 5 shows, the Doylestown algorithm maintained its superior detection of HCC as compared to AFP and actually increased the improvement in sensitivity over AFP alone. Increasing the cut-off of AFP to 100 ng/mL or the Doylestown algorithm to 0.75 decreased detection of HCC but once again, the Doylestown algorithm maintained its superior detection of HCC as compared to AFP.

**Table 5:** Sensitivity of AFP and the Doylestown algorithm at times before HCC diagnosis.

	Time prior to HCC diagnosis		
Marker	1–3 months (n = 104)	6–9 month (n = 52)	12 months (n = 35)
AFP (20 ng/mL)^**1**^	58%	30%	36%
Doylestown algorithm (0.50)^**2**^	71%	50%	50%
P value^3^	0.0550	0.0360	0.2250
Power^4^	0.5530	0.6749	0.5001
AFP (10 ng/mL) ^**5**^	68%	42%	54%
Doylestown algorithm (0.25) ^**6**^	87%	69%	82%
P value^3^	0.0029	0.0051	0.0166
power^4^	0.9170	0.8774	0.7918
AFP (100 ng/mL) ^**7**^	28%	12%	13%
Doylestown algorithm (0.75)^**8**^	44%	19%	31%
P value^3^	0.0185	0.021	0.0650
power^4^	0.9259	0.2569	0.5510

1) Sensitivity using the indicated cut-off (see below) of AFP at either 1–3 months, 6–9 months, or 12 months before HCC detection using a cut-off of 20 ng/mL.

2) Sensitivity of the Doylestown algorithm at either 1–3 months, 6–9 months, or 12 months before HCC detection using a cut-off of 0.50 output units.

3) P value between AFP and the Doylestown algorithm using a one-sided alternative hypothesis test.

4) Power level of this difference.

5) Sensitivity of AFP at either 1–3 months, 6–9 months, or 12 months before HCC detection using a cut-off of 10 ng/mL.

6) Sensitivity of the Doylestown algorithm at either 1–3 months, 6–9 months, or 12 months before HCC detection using a cut-off of 0.25 output units.

7) Sensitivity of AFP at either 1–3 months, 6–9 months, or 12 months before HCC detection using a cut-off of 100 ng/mL

8) Sensitivity of the Doylestown algorithm at either 1–3 months, 6–9 months, or 12 months before HCC detection using a cut-off of 0.75 output units.

### Performance of the Doylestown algorithm in those with recurrent HCC

Next, we compared AFP to the Doylestown algorithm for those patients who had HCC recurrence(n = 31). Clinical data was available at <3 months, 6 months, 9 months and 12 months prior to cancer recurrence. The mean size of the tumor at time of diagnosis was 1.8 cm (0.8–5.5 cm). A scatter plot of the three main components of the Doylestown algorithm (AFP, ALK and ALT) along with the output values for the Doylestown algorithm are shown in S5 Fig. The mean values of AFP, ALK and ALT are shown in Table 6.

**Table 6:** Individual component and the Doylestown algorithm at different time-points before HCC recurrence.

Time before HCC detected ^1^	<1–3 months	6 months	9 months	12 months	*P value*^*7*^
Size of tumor at time of detection,cm^**2**^	1.8 (0.8–5.5)				
ALK (range,U/L) ^**3**^	136.9 (34–570)	111.8 (30–191)	117.4 (38–205)	130.1 (38–360)	0.746
ALT (rangeU/L) ^**4**^	66.3 (11–309)	69.8 (16–229)	54.5 (13–166)	56.5 (8–268)	0.8576
AFP (range,ng/mL) ^**5**^	102 (1–1618)	23.6 (1.6–84.5)	15.6 (1.5–64.4)	14.5 (1.7–92.7)	0.184
Doylestown algorithm (range) ^**6**^	0.66 (0.08–1)	0.62 (0.12–1)	0.56 (0.12–0.96)	0.59 (.09–0.97)	0.5180

1) Time before detection of HCC, either <1–3 months prior, 6 months prior, 9 months prior or 12 months prior.

2) Mean size of tumor (in cm) at time of detection with the range provided.

3) Mean level of alkaline phosphatase with the range indicated (in U/L).

4) Mean level of alanine aminotransferase with the range indicated (in U/L).

5) Mean level of alpha-fetoprotein (AFP) with the range indicated (in ng/mL).

6) Mean level of the Doylestown algorithm with the range indicated.

7) Linear mixed effects model.

Using individual patient clinical values from patients 12 months prior to the confirmed diagnosis of HCC recurrence by imaging or biopsy, AFP and the Doylestown algorithm identified 18% and 59% of the recurrent HCC respectively (Table 7, p = 0.0021). At 9 months prior to HCC recurrence, AFP detected 29% of individuals as having HCC and the Doylestown algorithm increased this to 57% (p = 0.0202). Similar results were obtained at 6 months prior to recurrence; AFP and the Doylestown algorithm identified 27% and 64% of the HCC respectively (p = 0.0024). Within 3 months of HCC recurrence, AFP correctly identified HCC in 47% and the Doylestown algorithm accuracy increased to 67% (p = 0.0999). The impact of altering the cut-off for AFP or the Doylestown algorithm output value on HCC detection is shown in Table 7. As this table shows, decreasing the cut-off to 10 ng/mL for AFP or to 0.25 for the Doylestown algorithm increased the detection of HCC for both markers. However, as Table 7 shows, the Doylestown algorithm maintained it superior detection of HCC as compared to AFP. Increasing the cut-off of AFP to 100 ng/mL or the Doylestown algorithm to 0.75 decreased detection of HCC but once again, the Doylestown algorithm maintained it superior detection of HCC as compared to AFP.

**Table 7:** Sensitivity of AFP and the Doylestown algorithm at times before HCC recurrence.

Analyte	<1–3 months	6 month	9 months	12 months
AFP (20 ng/mL) ^**1**^	47%	27%	29%	18%
Doylestown algorithm (0.50) ^**2**^	67%	64%	57%	59%
P value^**3**^	0.0999	0.0024	0.0202	0.0021
Power^**4**^	0.4829	0.9121	0.7306	0.9615
AFP (10 ng/mL) ^**5**^	53%	55%	43%	32%
Doylestown algorithm (0.25) ^**6**^	78%	82%	81%	82%
P value^**3**^	0.0316	0.0137	0.0021	>0.0001
Power^**4**^	0.6768	0.7565	0.9383	0.9994
AFP (100 ng/mL) ^**7**^	14%	0%	0%	0%
Doylestown algorithm (0.75) ^**8**^	53%	36%	29%	32%
P value^**3**^	0.0014	0.0004	0.0019	0.0009
Power^**4**^	0.9604	0.9996	0.9976	0.9989

1) Sensitivity of AFP at either <1–3 months, 6 months, 9 month or 12 months before HCC detection using a cut-off of 20 ng/mL.

2) Sensitivity of the Doylestown algorithm at either <1–3 months, 6 months, 9 month or 12 months before HCC recurrence using a cut-off of 0.50 output units.

3) P value between AFP and the Doylestown algorithm using a one-sided alternative hypothesis test.

4) Power level of this difference.

5) Sensitivity of AFP at either <1–3 months, 6 months, 9 month or 12 months before HCC recurrence using a cut-off of 10 ng/mL.

6) Sensitivity of the Doylestown algorithm at either <<1–3 months, 6 months, 9 month or 12 months before HCC recurrence using a cut-off of 0.25 output units.

7) Sensitivity of AFP at either <1–3 months, 6 months, 9 month or 12 months before HCC recurrence using a cut-off of 100 ng/mL

8) Sensitivity of the Doylestown algorithm at either <1–3 months, 6 months, 9 month or 12 months before HCC recurrence using a cut-off of 0.75 output units.

## Discussion

The development and validation of the Doylestown algorithm in predicting HCC had been done using measurements of the variables collected, at a single time, from case-control or nested case-control studies[9]. Here, we presented a retrospective, longitudinal study applying the Doylestown algorithm to serial time points in patients with chronic liver diseases with and without HCC to demonstrate the robustness of the algorithm. The inclusion of control patients, with chronic liver diseases without liver cirrhosis or HCC confirmed the specificity of this algorithm. It was essential to determine that the fluctuations in the key components of the algorithm (such as an individual’s AFP, ALK and ALT values) do not adversely impact the performance of the Doylestown algorithm and falsely identify those who do not have HCC. The application of the Doylestown algorithm did lead to false positive results in 2 patients. Both patients were older and had values close to the 0.5 cut-off. The 82 year old patient had normal AFP, ALK and ALT levels, the advanced age accounted for the false positive value of the Doylestown algorithm in these 2 cases. The algorithm was originally developed using a cohort of patients younger than 70 years old. Thus, this algorithm may not be accurate for those > 70 years of age and we are currently developing novel biomarkers and algorithms for those patient without cirrhosis but with chronic HBV infections.

The Doylestown algorithm, which combines AFP with clinical values increased the detection of HCC as compared to AFP alone at all time points examined. Using a fixed cut-off of 20 ng/mL for AFP and 0.5 output units for the Doylestown algorithm resulted in a 13% increased at the closest time point to HCC detection (<1 month), a 20% increase at the 6–9 month-time point and a 14% increase at the 12 month-time point (Table 7). This improvement in cancer predication also applied to those with HCC recurrence (Table 7). Indeed, at 12 months prior to the detection of recurrent HCC, the Doylestown algorithm increased the sensitivity of AFP alone by over 3 fold. It is noted that one patient with a large tumor at recurrence (5.5 cm lesion) was AFP negative (AFP value of 6.2 ng/mL) at the time of diagnosis, which may explain this person’s late diagnosis. The Doylestown algorithm values for this individual were 0.5 at the 12 month time period (with an AFP of 6.5 ng/mL) and rose to 0.9 at the time of diagnosis. Importantly, as we have shown here and in our previous report[9], the specificity of the Doylestown algorithm was similar or better then AFP. Together, these data suggest that the Doylestown algorithm enhances the prediction of HCC compared to AFP alone with increased sensitivity and similar specificity.

A limitation of the current study is the sample size that was available. For example, clinical data was only available from 35 patients at a time of 12 months prior to HCC diagnosis. A larger study with time points up to 2-years prior to HCC diagnosis will have to be performed to fully demonstrate the true benefit of the use of this algorithm for HCC surveillance. It is noted that in no case did the use of the Doylestown algorithm reduce the number of patients detected as compared to AFP, suggesting again, what was observed in our previous study, that the use of this algorithm does not impart any harm.

It is noted that in the case of patients with recurrent cancer, the sample size and difference between AFP and the Doylestown algorithm was large enough to provide >85% power and highlight the benefit in the use of the algorithm as opposed to AFP in this setting. However, future work will need to be performed in those who do not have re-occurrent disease. Such a study will require careful analysis and the inclusion of patients with MRI/CT-scan proof of no HCC at a time point of 12–24 months post last data point.

This study demonstrated that the Doylestown algorithm, by using readily available clinical parameters, is superior to AFP alone in predicating accurately the development of initial and recurrent HCC among patients with chronic liver diseases. While this algorithm is practical and easy to adopt in routine clinical use, it is important to emphasize that the current algorithm can be further complemented and improved with novel biomarkers to achieve early detection of hepatocellular carcinoma.

## Supporting information

S1 Fig Scatter plot of AFP and output value of the Doylestown algorithm in those in the control group and in those with HCC. A) AFP levels in the control and HCC group. X-axis represents the group and Y-axis is AFP level (ng/mL). B) Output values of the Doylestown Algorithm in the control and HCC group. X-axis represents the group and Y-axis is the output value from equation. The mean and 95% confidence interval for the mean are indicated.
(TIFF)

S2 Fig Scatter plot of ALK, ALT AFP and the output value of the Doylestown algorithm in control patients as a function of time. A) Alkaline phosphatase (ALK), B) Alanine aminotransferase (ALT), C) alpha feto protein (AFP) or D) Output values from the Doylestown algorithm in control patients at time 0, 6 months prior or 12 months prior. ALK and ALT levels are in IU/L and AFP is in ng/mL. The mean and 95% confidence interval for the mean are indicated.
(TIFF)

S3 Fig Scatter plot of ALK, ALT AFP and the output value of the Doylestown algorithm in HCC patients as a function of time. A) Alkaline phosphatase (ALK), B) Alanine aminotransferase (ALT), C) Log alpha feto protein (AFP) or D) Output values from the Doylestown algorithm in control patients at time 1–3 months before HCC detection, 6–9 months prior or 12 months prior to HCC detection. ALK and ALT levels are in IU/L and AFP is in ng/mL. The mean and 95% confidence interval for the mean are indicated.
(TIFF)

S4 Fig Receiver operator curves for AFP or the Doylestown Algorithm at a time of A) 1–3 before HCC diagnosis B) 6 to 9 months before HCC diagnosis or C) 12 months prior to HCC diagnosis.
(TIFF)

S5 Fig Scatter plot of ALK, ALT AFP and the output value of the Doylestown algorithm in patients with recurrent HCC as a function of time. A) Alkaline phosphatase (ALK), B) Alanine aminotransferase (ALT), C) alpha feto protein (AFP) or D) Output values from the Doylestown algorithm in control patients at time <1 month before HCC detection, 3–6 months prior or 9–12 months prior to HCC detection. ALK and ALT levels are in IU/L and AFP is in ng/mL. The mean and 95% confidence interval for the mean are indicated.
(TIFF)

S1 File Combined data for all samples. Excel sheets containing raw data for all control, HCC, and recurrent HCC samples used in this study.
(XLSX)
