# Correction: Application of the Doylestown algorithm for the early detection of hepatocellular carcinoma

**DOI:** 10.1371/journal.pone.0206835

**Published:** 2018-10-29

**Authors:** Anand S. Mehta, Daryl T.-Y. Lau, Mengjun Wang, Aysha Aslam, Bilal Nasir, Asad Javaid, Mugilan Poongkunran, Timothy M. Block

The fourth author's name is spelled incorrectly. The correct name is: Aysha Aslam. The correct citation is: Mehta AS, Lau DT-Y, Wang M, Aslam A, Nasir B, Javaid A, et al. (2018) Application of the Doylestown algorithm for the early detection of hepatocellular carcinoma. PLoS ONE 13(8): e0203149. https://doi.org/10.1371/journal.pone.0203149
