# Supplementary figures and images for: Application of the Doylestown algorithm for the early detection of hepatocellular carcinoma
Source: PLoS One. 2018 Aug 31;13(8):e0203149. doi: 10.1371/journal.pone.0203149 (PMC6118370; doi:10.1371/journal.pone.0203149)

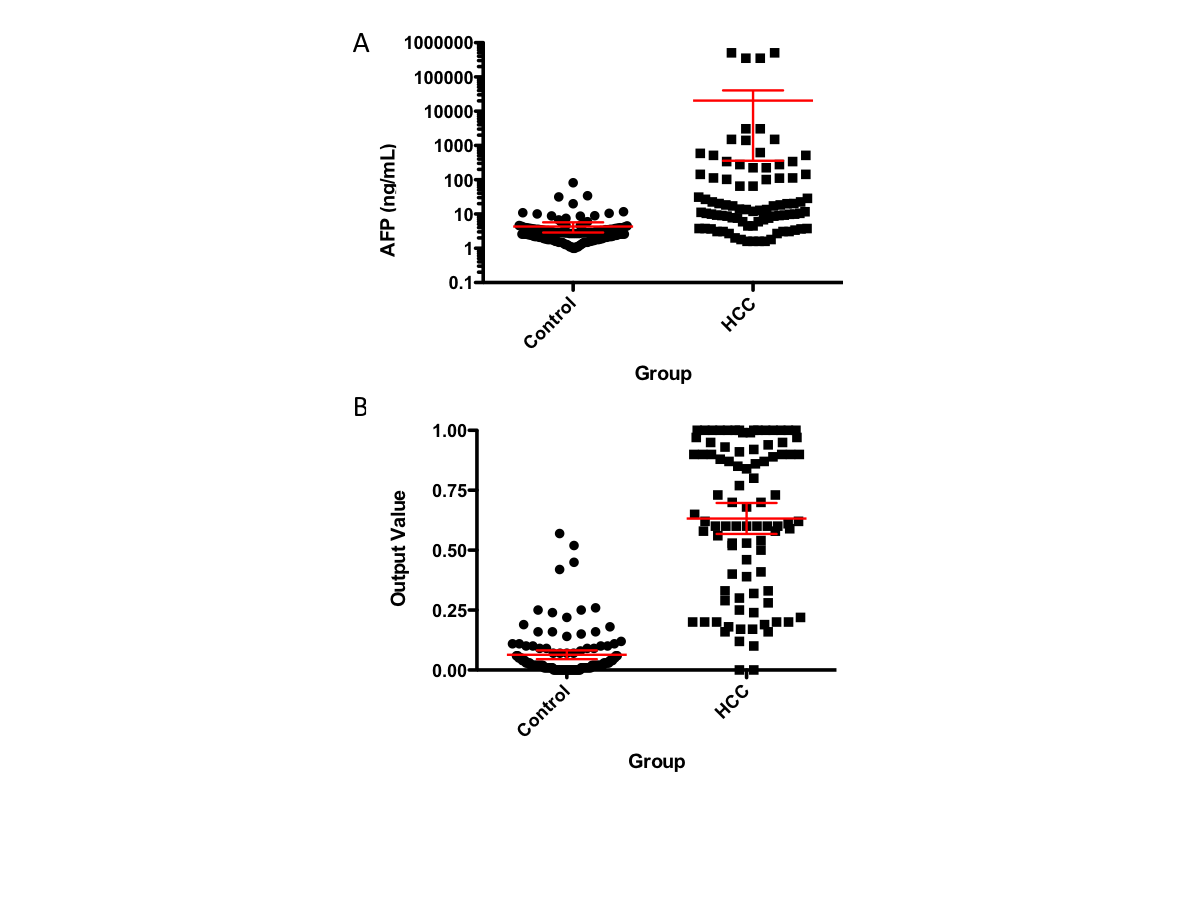

Supplement: S1 Fig — A) AFP levels in the control and HCC group. X-axis represents the group and Y-axis is AFP level (ng/mL). B) Output values of the Doylestown Algorithm in the control and HCC group. X-axis represents the group and Y-axis is the output value from equation. The mean and 95% confidence interval for the mean are indicated. (TIFF) [file pone.0203149.s001.tiff]

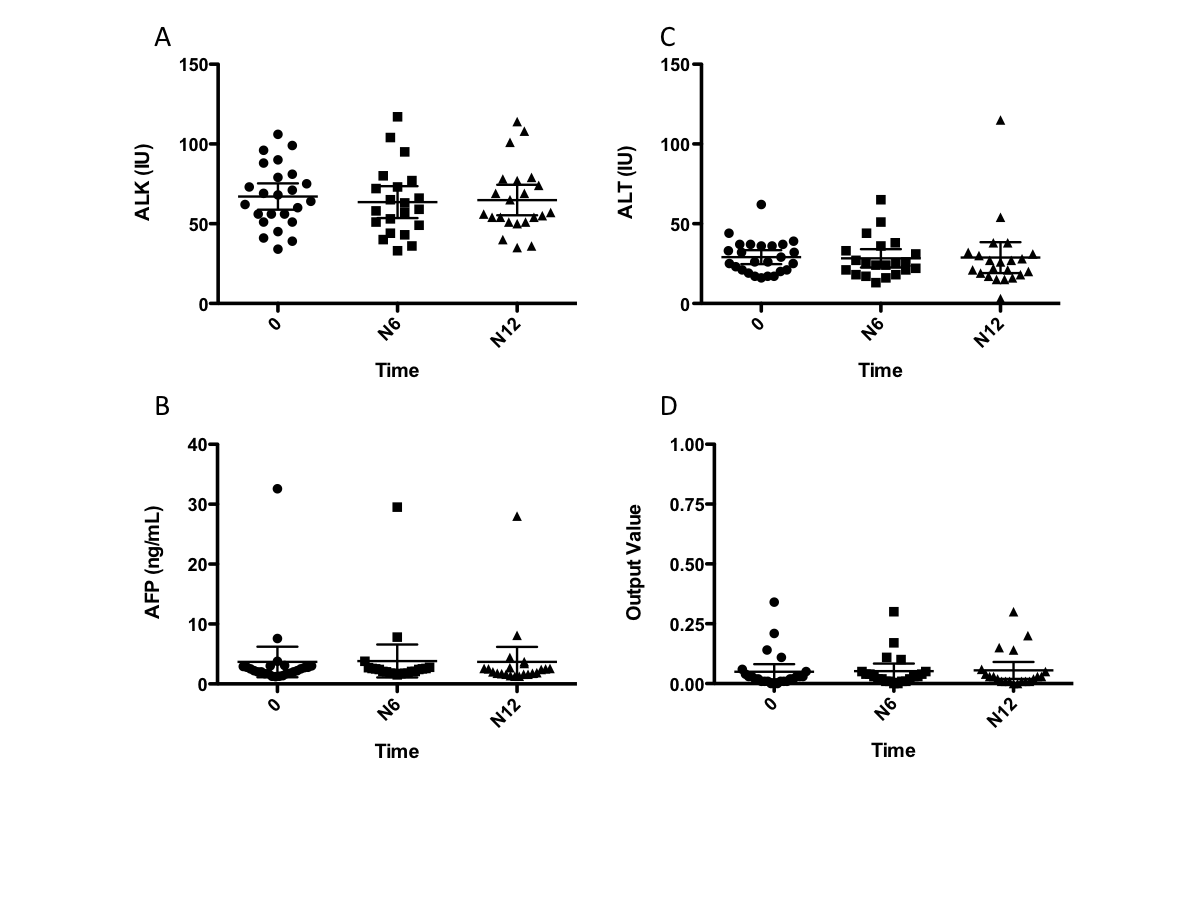

Supplement: S2 Fig — A) Alkaline phosphatase (ALK), B) Alanine aminotransferase (ALT), C) alpha feto protein (AFP) or D) Output values from the Doylestown algorithm in control patients at time 0, 6 months prior or 12 months prior. ALK and ALT levels are in IU/L and AFP is in ng/mL. The mean and 95% confidence interval for the mean are indicated. (TIFF) [file pone.0203149.s002.tiff]

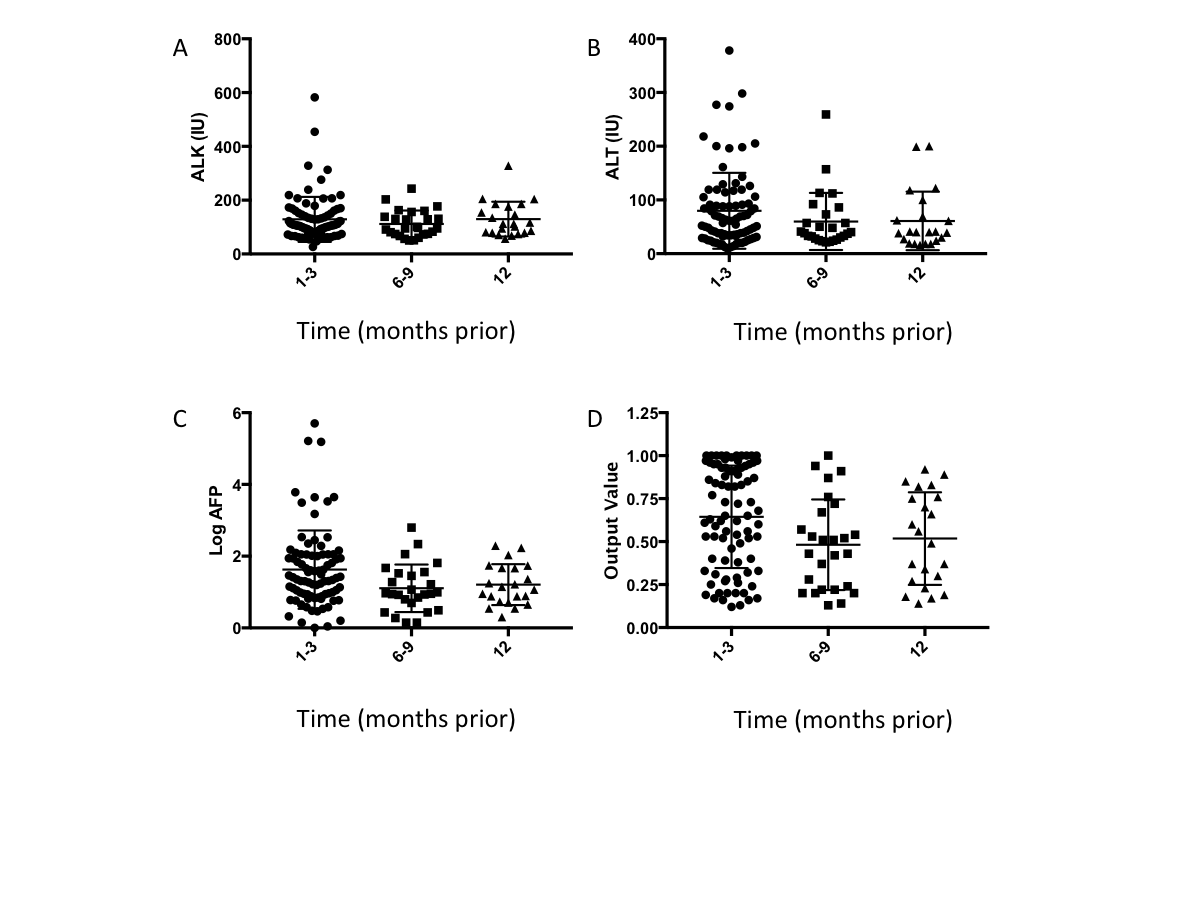

Supplement: S3 Fig — A) Alkaline phosphatase (ALK), B) Alanine aminotransferase (ALT), C) Log alpha feto protein (AFP) or D) Output values from the Doylestown algorithm in control patients at time 1–3 months before HCC detection, 6–9 months prior or 12 months prior to HCC detection. ALK and ALT levels are in IU/L and AFP is in ng/mL. The mean and 95% confidence interval for the mean are indicated. (TIFF) [file pone.0203149.s003.tiff]

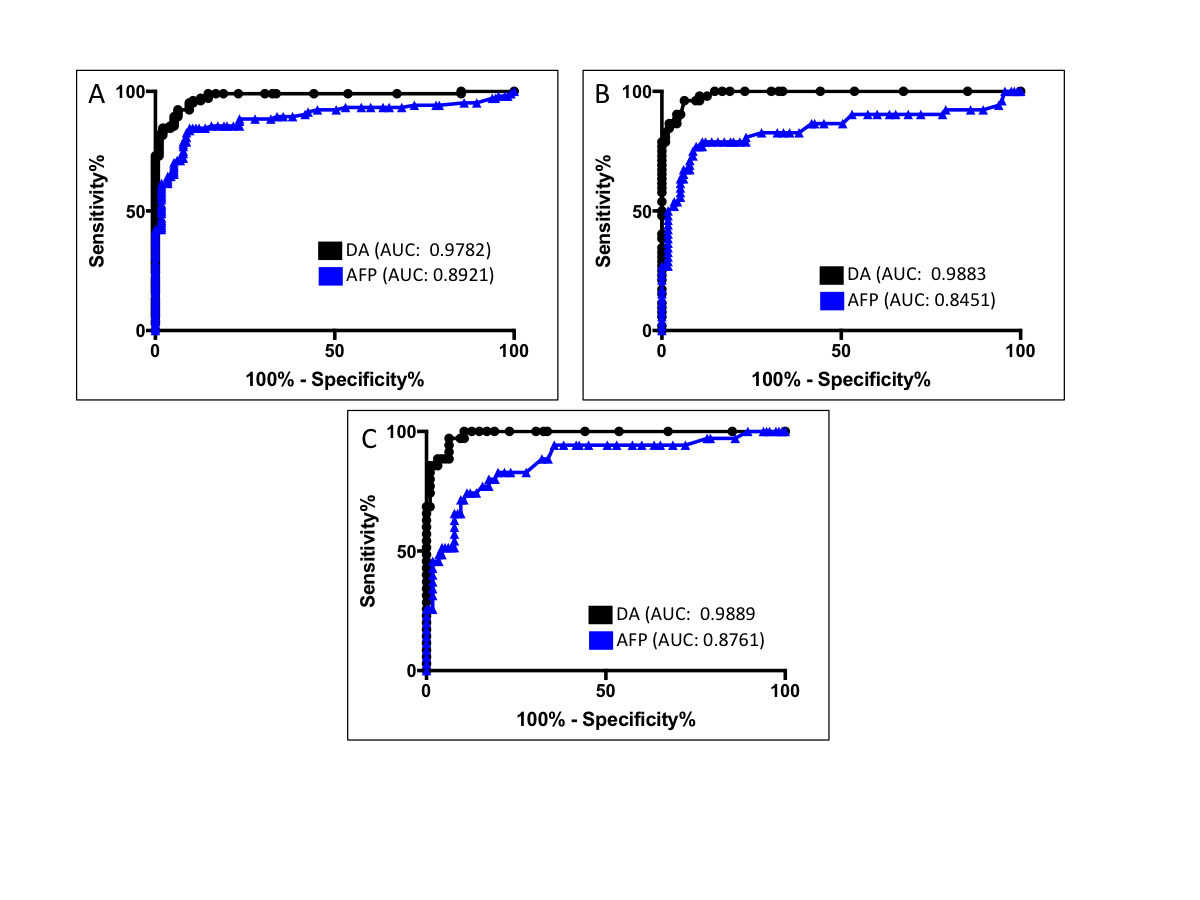

Supplement: S4 Fig — Receiver operator curves for AFP or the Doylestown Algorithm at a time of A) 1–3 before HCC diagnosis B) 6 to 9 months before HCC diagnosis or C) 12 months prior to HCC diagnosis. (TIFF) [file pone.0203149.s004.tiff]

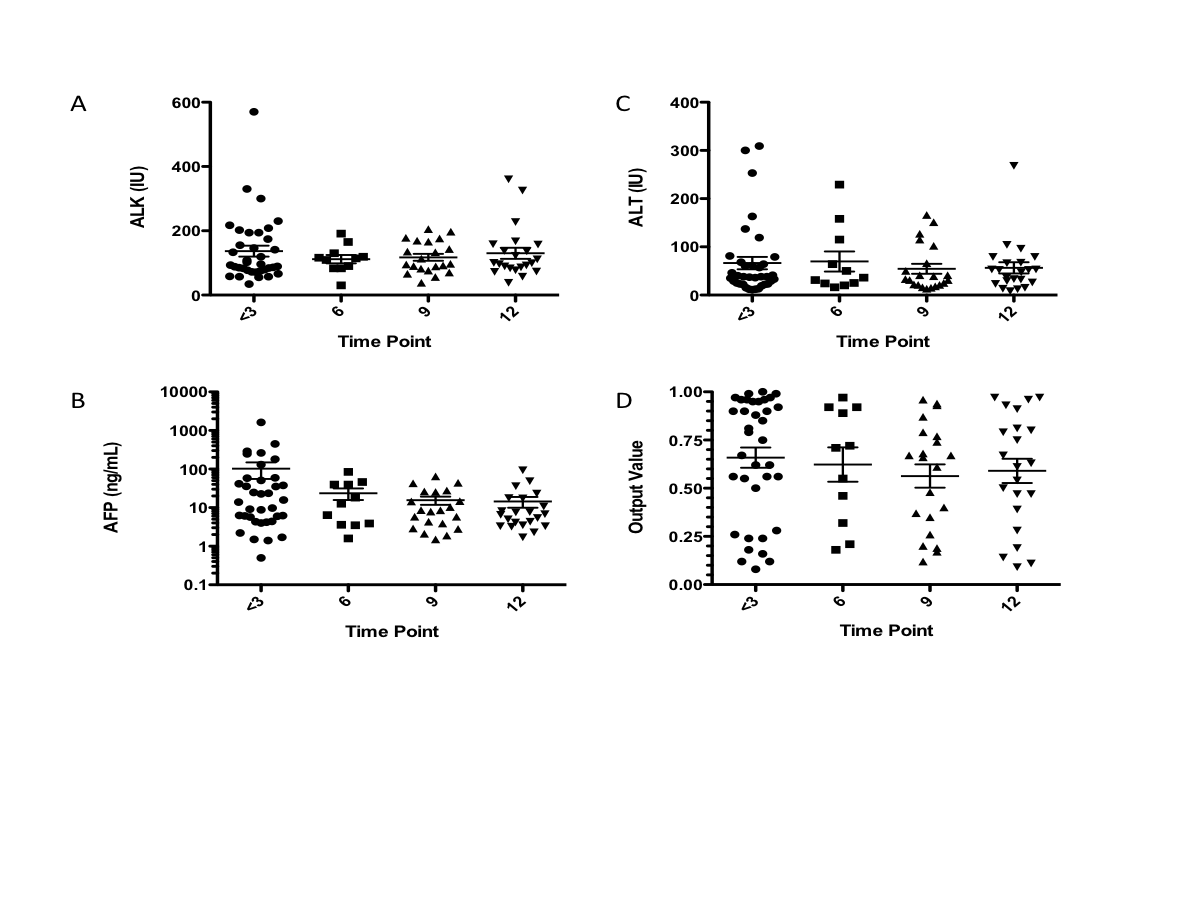

Supplement: S5 Fig — A) Alkaline phosphatase (ALK), B) Alanine aminotransferase (ALT), C) alpha feto protein (AFP) or D) Output values from the Doylestown algorithm in control patients at time <1 month before HCC detection, 3–6 months prior or 9–12 months prior to HCC detection. ALK and ALT levels are in IU/L and AFP is in ng/mL. The mean and 95% confidence interval for the mean are indicated. (TIFF) [file pone.0203149.s005.tiff]
